# Hot Topics in Nutrition and Obesity: Integrating Epidemiology, Mechanisms, and Therapeutic Perspectives

**DOI:** 10.3390/nu18101595

**Published:** 2026-05-18

**Authors:** Victoria Catalán, Javier Gómez-Ambrosi

**Affiliations:** 1 Metabolic Research Laboratory, Clínica Universidad de Navarra, 31008 Pamplona, Spain; 2 Institute for Nutrition & Health, University of Navarra, 31008 Pamplona, Spain; 3 Centro de Investigación Biomédica en Red-Fisiopatología de la Obesidad y Nutrición (CIBEROBN), Instituto de Salud Carlos III, 31008 Pamplona, Spain; 4 Obesity and Adipobiology Group, Instituto de Investigación Sanitaria de Navarra (IdiSNA), 31008 Pamplona, Spain

## 1. Introduction: The Global Burden of Obesity

Obesity remains one of the most pressing global public health challenges, contributing significantly to the development of metabolic disorders, including type 2 diabetes, cardiovascular disease, dyslipidemia, metabolic dysfunction-associated steatotic liver disease (MASLD/MASH), and certain types of cancer [1,2]. Despite substantial advances in our understanding of obesity biology and the emergence of innovative therapeutic strategies, its global prevalence continues to rise, underscoring the need for comprehensive and multidisciplinary approaches.

On the 15th anniversary of *Nutrients*, this Special Issue, “Hot Topics on Nutrition and Obesity 2024”, was launched to provide an updated overview of key developments in the field. The aim was to gather high-quality contributions spanning nutritional epidemiology, mechanistic research, clinical and lifestyle interventions, and emerging technologies, reflecting the multifactorial nature of obesity. This collection includes eight articles—seven original research papers and one review—that collectively illustrate the complexity of obesity from population-level determinants to molecular mechanisms and therapeutic perspectives.

## 2. Epidemiological Insights and Population-Level Trends

Understanding the distribution and determinants of obesity at the population level is essential for informing effective prevention strategies. Epidemiological studies provide critical insights into prevalence patterns, temporal trends, and sociodemographic disparities, helping to identify high-risk groups and guide targeted public health interventions [3,4]. At the population level, the study by Traczyk et al. (contribution 4) provides a comprehensive analysis of the prevalence of overweight, general obesity, and abdominal obesity in a large sample of Polish adults, based on data from the National Health Program (2016–2020). Importantly, the authors not only quantify the burden of excess body weight but also examine its distribution across sociodemographic groups, revealing disparities associated with age, sex, and socioeconomic status. These findings reinforce the uneven distribution of obesity and highlight the need for targeted public health strategies.

Complementing these epidemiological insights, Jakše et al. (contribution 5) present a longitudinal assessment of body composition changes in Slovene adults over a two-year period. By incorporating both BMI and body fat percentage classifications, they demonstrate that reliance on BMI alone may underestimate true adiposity and related health risks. The longitudinal design further allows the authors to capture dynamic changes in body composition, offering valuable evidence on the progression of obesity and the importance of continuous monitoring.

## 3. Advances in Body Composition Assessment

Accurate assessment of body composition is fundamental for the proper diagnosis and management of obesity [5,6]. Beyond conventional anthropometric indices, emerging methodologies allow for a more precise evaluation of adiposity and its distribution, particularly in populations where standard measures may be limited or misleading [7,8,9,10]. The importance of accurate body composition assessment is further emphasized in the study by Bagińska-Chyży and Korzeniecka-Kozerska (contribution 6), which focuses on children with spina bifida. In this population, standard anthropometric measures are often inadequate due to altered growth patterns and body proportions. The authors demonstrate that bioelectrical impedance analysis provides a more reliable tool for assessing adiposity and guiding weight management interventions, underscoring the need for tailored approaches in vulnerable and clinically complex populations.

## 4. Mechanistic Insights into Obesity and Metabolic Dysfunction

Elucidating the biological mechanisms underlying obesity is key to understanding its associated complications and identifying novel therapeutic targets [11,12]. Experimental and translational studies continue to uncover the complex interactions between the dietary components, cellular processes, and organ systems involved in metabolic dysfunction. The mechanistic studies in this Special Issue shed light on the biological underpinnings of obesity and its complications. Sivasubramanian et al. (contribution 7) investigate the effects of palmitic acid, a common saturated fatty acid, on human brainstem astrocytes. Their findings reveal that it induces oxidative stress and cellular senescence while impairing glutamate transporter expression, suggesting a potential link between dietary fat intake, neuroinflammation, and obesity-associated sympathetic overactivity. This work contributes to a growing body of evidence implicating central nervous system mechanisms in obesity-related cardiometabolic dysfunction.

In a complementary experimental study, Kang and Cao (contribution 3) explore the therapeutic potential of ursolic acid in a high-fat diet-induced obesity model. Their results show that ursolic acid supplementation improves muscle strength, enhances lipid metabolism, and reduces hepatic steatosis. These findings are particularly relevant in the context of sarcopenic obesity and metabolic liver disease, highlighting the potential of naturally occurring bioactive compounds as adjunctive treatments.

## 5. Inflammation and Immune–Nutritional Interactions

Chronic low-grade inflammation is a hallmark of obesity and a major contributor to its metabolic consequences [13,14,15]. Investigating the interplay between nutritional status and immune responses provides valuable insights into disease progression and highlights potential biomarkers and intervention targets. The role of inflammation as a key mediator of obesity-related complications is further explored by Uzun et al. (contribution 2), who examine the relationship between nutritional status and systemic immune–inflammation indices across BMI categories. By integrating multiple validated nutritional and inflammatory markers, the authors demonstrate a clear association between worsening nutritional status, increased adiposity, and heightened systemic inflammation. This reinforces the concept of obesity as a chronic low-grade inflammatory state and highlights the value of composite indices in risk stratification.

## 6. Gut Microbiota and Emerging Therapeutic Targets

The gut microbiota has emerged as a central player in the regulation of host metabolism and energy balance [16,17,18]. Advances in this field are shedding light on how diet–microbe interactions influence obesity development and how microbiota-targeted strategies may offer innovative therapeutic opportunities. Positioned at the interface of diet, microbiota, and metabolic health, the review by Yi et al. (contribution 8) provides a comprehensive synthesis of microbiota-based interventions in the context of high-fat diet-induced metabolic alterations. The authors discuss how changes in gut microbial composition, bile acid metabolism, and host–microbe interactions contribute to obesity pathogenesis. Importantly, they also evaluate potential therapeutic strategies aimed at restoring microbial balance, offering insights into a rapidly evolving and promising area of research.

## 7. Psychosocial and Behavioral Determinants of Obesity

Obesity is not only driven by biological factors but is also strongly influenced by behavioral and psychosocial determinants [19,20,21]. Understanding the role of stress, emotional regulation, and lifestyle behaviors is crucial for developing comprehensive and sustainable prevention and intervention strategies. The psychosocial dimension of obesity is addressed by Głąbska et al. (contribution 1), who investigate the influence of stress and emotional eating on body mass in a population of Polish female adolescents. Their findings highlight the significant role of emotional and behavioral factors in shaping dietary habits and weight outcomes. The study underscores the importance of early-life interventions that incorporate psychological support and behavioral strategies to prevent the development of obesity.

## 8. Conclusions

Taken together, the contributions in this collection underscore the multifactorial nature of obesity, encompassing biological, behavioral, and environmental determinants. They highlight the need for integrated approaches that combine precise phenotyping, mechanistic understanding, and targeted interventions. Future research should aim to elucidate causal relationships, improve the precision of nutritional assessments, and develop personalized and scalable interventions. In particular, emerging fields such as nutrigenomics, digital health technologies, and microbiome research offer promising avenues for innovation.

In conclusion, this Special Issue provides a comprehensive overview of current “hot topics” in nutrition and obesity. By bringing together diverse lines of evidence, it contributes to advancing our understanding of this complex condition and supports the development of more effective strategies to address the ongoing obesity pandemic.
